# An Evaluation of 3D-Printed Materials’ Structural Properties Using Active Infrared Thermography and Deep Neural Networks Trained on the Numerical Data

**DOI:** 10.3390/ma15103727

**Published:** 2022-05-23

**Authors:** Barbara Szymanik

**Affiliations:** Center for Electromagnetic Fields Engineering and High-Frequency Techniques, Faculty of Electrical Engineering, West Pomeranian University of Technology, Szczecin, Sikorskiego 37, 70-313 Szczecin, Poland; szymanik@zut.edu.pl

**Keywords:** active thermography, deep learning, numerical modeling, LSTM neural networks, 3D-printed structure quality

## Abstract

This article describes an approach to evaluating the structural properties of samples manufactured through 3D printing via active infrared thermography. The mentioned technique was used to test the PETG sample, using halogen lamps as an excitation source. First, a simplified, general numerical model of the phenomenon was prepared; then, the obtained data were used in a process of the deep neural network training. Finally, the network trained in this manner was used for the material evaluation on the basis of the original experimental data. The described methodology allows for the automated assessment of the structural state of 3D−printed materials. The usage of a generalized model is an innovative method that allows for greater product assessment flexibility.

## 1. Introduction

### 1.1. Quality Control of 3D−Printed Materials

The use of additive manufacturing (AM), otherwise known as 3D printing, is now prevalent in many industries. Early on, printed structures were mostly used in the design and development of the devices’ prototypes. However, the progression of 3D printing techniques has led to the fact that these structures are becoming more common in final product production [1,2]. As a result of the wide range of materials available for AM, printed elements are present in advanced industrial settings, including but not limited to the medical (for building blood vessels or constructing low−cost prosthetic parts), the architectural and the automotive industries [3,4,5,6,7]. It is crucial to assess the quality of these materials given their widespread use. The process of additive manufacturing can be controlled at all stages—the preparation (examination of the quality of the feedstock material), production (control of the printing process), and evaluation of the end−product. Herein, we focus on quality control in the final stage of 3D printing production [8,9,10,11].

A great deal of work has been done in the previous decade to evaluate the quality of materials produced via additive manufacturing, and a variety of approaches have been introduced. Non−destructive testing (NDT) procedures are one of the most important methodologies in the field of materials science [12,13,14,15]. The use of NDT technologies, in particular, allows for the identification and characterization of surface as well as internal defects of materials without the need for any physical interactions, such as cutting or modifying the substance. Along with these benefits, NDT techniques are designed to be a cost−effective approach of system quality management. Non−destructive evaluations of final AM products include the evaluation of dimensional correctness and surface quality, the evaluation of internal structure, and the detection of defects. The most widely used methods for evaluating the surface quality of printed items are vision [16], microscopy [17], and laser profilometry [18,19]. The internal structure of 3D printing, including the detection of subsurface flaws, is mostly examined using computed tomography [20] and ultrasound [21,22].

In this article, one of the non−destructive testing (NDT) methods—active infrared thermography (AIT)—was utilized to examine the printed structure on a qualitative level [23,24]. An external energy source (here, halogen lamps) was employed to induce a thermal gradient within the test sample in this procedure. A thermal imaging camera was used to monitor the variation in temperature on the surface of the sample. Divergences (warmer or colder regions) in the observed temperature distribution indicate heterogeneities within the analyzed structure. The evaluated 3D−printed samples were designed in a form of a basic structure—a flat cuboid with imprinted holes of varying sizes and depths, imitating the object’s structural degradation.

### 1.2. Deep Neural Networks in Nondestructive Testing of Materials

The nondestructive testing of materials encompasses both the qualitative and quantitative evaluation of the materials under test. It is incredibly beneficial to apply a variety of machine learning methods for this assignment. A large number of machine learning approaches are based on feature extraction performed by experts [25,26]. As a result, the quality of the features (of the stated phenomenon or qualities) stored in the database has a significant impact on the efficacy of classification or clustering algorithms, making the extraction process crucial. To meet this challenge, the recent rapid growth of complex multi−layered artificial neural networks (ANNs) can be observed. In general, artificial neural networks (ANNs) attempt to mimic the human learning process in order to independently learn features [27,28]. As a result, there is no requirement for feature extraction based on human judgment in these methods.

In this article, to examine the state of the material, an automated defects detection technique based on neural network models was developed [29,30,31]. An investigation of the signals received during an AIT examination in the form of time–temperature characteristics is carried out in this study. Consequently, the primary purpose of this study is data categorization on the basis of one−dimensional sequences. The manual analysis of such sequences may be difficult due to an overwhelming quantity of data, making it difficult to identify patterns. Thus, the challenge of identifying patterns in time sequences in order to forecast the predicted output may be accomplished via the use of artificial intelligence systems. Recurrent neural networks (RNNs), as well as their modifications, are one of the most successful technologies accessible in this situation [32,33].

As is generally known, a recurrent neural network is a kind of neural network that is optimized for processing time−dependent data sequences. RNNs are often preferred over other types of neural networks for applications that require sequential inputs, such as voice and language recognition. RNNs are referred to as recurrent neural networks because they complete the same job for each element in a sequence, with the output being dependent on the results of the previous computations in the sequence. Gradient calculation in RNNs is accomplished by executing a forward propagation pass followed by a backward propagation pass. Back−propagation through time (BPTT) is the method used here. Because the parameters are shared by all time steps in the network, the gradient at each output is determined by both the current and prior time steps’ computations. Although the RNN is a simple and strong model in theory, it is difficult to train well in reality [34,35]. The vanishing gradient and exploding gradient difficulties are two of the key reasons why this model is so problematic [36,37]. When utilizing BPTT for training, gradients must pass from the last cell to the first cell. The product of these gradients might either be 0 or exponentially grow. The issue of exploding gradients refers to the substantial growth in the gradient’s norm during training. When long−term components travel exponentially quickly to norm 0, the vanishing gradients issue occurs, making it hard for the model to learn connection between temporally distant events. Whereas the exploding gradient problem may be resolved relatively quickly utilizing techniques such as gradient clipping, the vanishing gradient issue remains a significant concern when employing an RNN. However, by using special modifications of recurrent networks such as long short−term memory (LSTM), gated recurrent units (GRUs), and residual networks (ResNets), this limitation may be solved [38,39]. Considering the above, it was determined in this article to employ LSTM networks to process the data sequences received from thermal imaging of 3D−printed samples.

### 1.3. Novelty and Significance of the Research

A key problem that occurs when utilizing neural networks to analyze diverse types of data is the training set selection. Extracting such a collection from the same data set, which is then assessed by a trained network, is the most often employed approach. In our previous work, we have shown that, for this purpose, the convolutional neural networks may be used [40], and other researchers have worked on a similar problem [41]. This results in the obvious issue of a network adjusting to a single−use case, rendering it very inflexible and unsuited for jobs requiring some automation. This issue may be resolved by the use of numerical modeling. If the numerical model generated accurately represents the true behavior of the system, we may be certain that the network trained on such prepared numerical data will be capable of evaluating real, experimental data. The most often utilized approach is to model the complete tested system, which, once again, may result in an overly adapted network to the tested system. We have tested this approach in our previous work, proving its effectivity [42]. However, we suggest a more general approach in this work: the development of a collection of models, each of which represents a small piece of the tested sample, containing one structural flaw and a chosen section of the surrounding region. We shall demonstrate that such a solution enables effective assessment of the material under test and improves the flexibility of the resulting network.

### 1.4. Organization of the Paper

The paper is structured as follows: the introduction discusses the non−destructive testing of AM materials and the artificial intelligence methods used to evaluate them, with a special emphasis on LSTM neural networks. The materials under investigation, as well as the experimental method employed—activated infrared thermography—are discussed in Section 2. As part of Section 3, the assumptions and structure of a parametric set of generalized numerical models are discussed along with the numerical model optimization, as well as the presentation of numerical findings and comparisons of numerical and experimental results. Section 4 discusses data processing techniques and the construction of a training data set for the neural network. The architecture of the suggested neural network and the results of the evaluation are presented in Section 5. The paper is summarized and concluded in Section 6.

## 2. Materials and Methods

The primary objective of this study was to create a method for analyzing real samples using neural networks trained on numerical data. The samples were initially tested experimentally. In this chapter, the utilized laboratory setup, test samples, and experimental methodologies will be described. In the subsequent sections of the study, it will be demonstrated how the tested system was reconstructed in a simplified numerical model.

### Experimental Techniques and Test Samples

**Figure 1:**
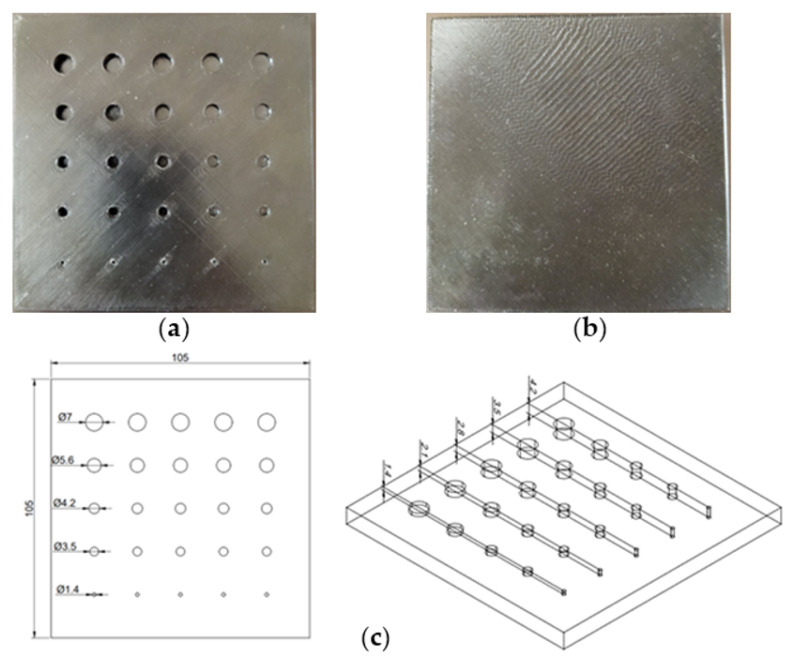
Experimental samples—(**a**) rear (heated) and (**b**) front (observed) side. (**c**) CAD model of printed structures (dimensions in [mm]).

**Figure 2:**
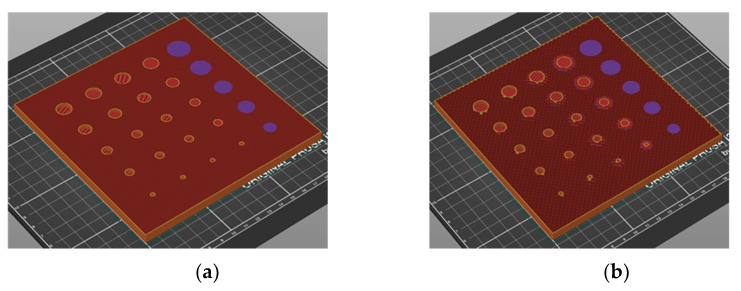
Visualization of the printing process for chosen layer (screenshot from the PrusaSlicer). (**a**) sample S1 with 100% infill, (**b**) sample S2 with 30% infill.

Two test samples were created using the Filament Fuse Fabrication (FFF) method, which is one of the most widely used 3D printing technologies currently available. We chose the broadly used polyethylene terephthalate glycol (PET−G) filament, which is known for being both durable and simple to print with. For the sample S1, the infill was adjusted to a maximum—100%—and for the sample S2, it was set to 30% with a rectangular infill pattern. Both samples were designed and printed as flat plates with dimensions of 105 × 105 × 7 mm, with a series of holes of sizes ranging from 1.4 to 7 mm and depths ranging from 1.4 to 4.2 mm. An example CAD model and a photograph of the sample are depicted in Figure 1. Moreover, Figure 2 presents the inner structure of the printed samples. The chosen layer of print is visualized here as an output from a G−code generating program—PrusaSlicer. The printing pattern is visible—rectangular for both of the samples—as well as the difference in the infill percentage and the increased infill in the area of the defects for the sample S2, which is crucial for the results of thermographic inspection. This issue will be discussed in detail in further sections.

**Figure 3:**
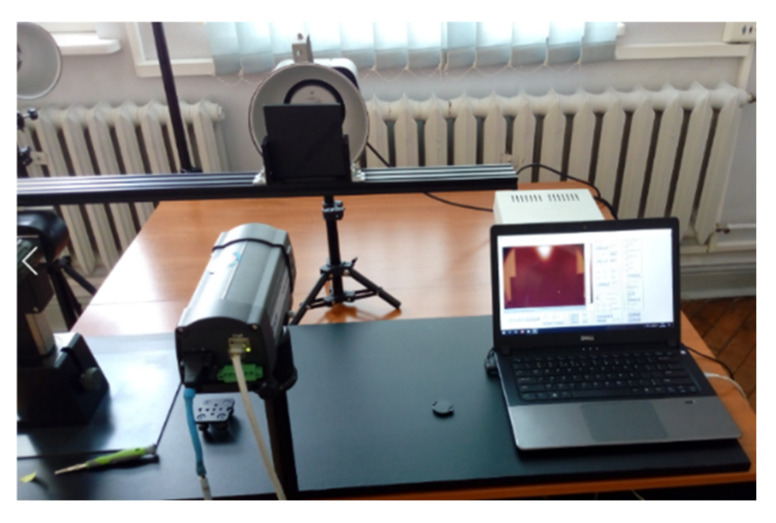
Photo of the experimental setup.

The material was examined using active infrared thermography (AIT), which used a halogen lamp as an excitation source. The transmission technique was utilized in this experiment: the material was heated from the back side while being monitored from the front. Because of the poor thermal conductivity of the material under test, a long step heating procedure was used. It was decided that the sample would be heated for 60 s and then naturally cooled for 300 s. During this time, a thermal imaging camera was used to record the temperature distribution on the front side of the sample at a rate of one frame per second. As a result of this experiment, 360 thermograms, ready for further processing, were produced. The experimental setup is illustrated in Figure 3.

## 3. Numerical Model

The numerical model for this study was created using the commercial software−Comsol Multiphysics. The calculations were performed utilizing Finite Element Method (FEM), with Comsol modules developed to evaluate heat transfer via conduction and radiation phenomena.

### 3.1. General Description of the Model

**Figure 4:**
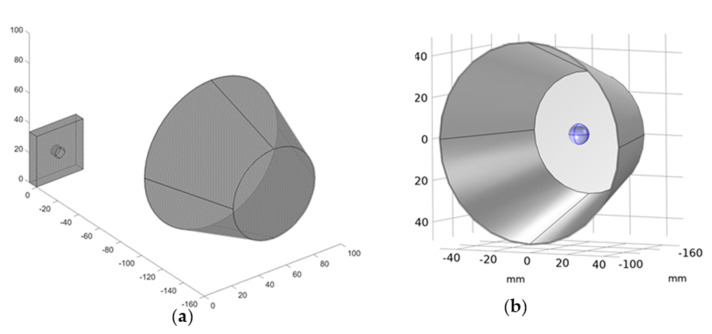
Numerical model geometry. (**a**) The view on the single−defect sample with the heat source, (**b**) detail of the modeled halogen lamp—heat reflecting, hollow cone with point heat source placed inside.

Figure 4 depicts the geometry of the numerical model created in Comsol. The heat source (in this experiment, a halogen lamp) was replicated using a hollow conical aluminum tube with a point heat source placed within at a 15 cm distance from the sample. Figure 4b depicts the source’s location. In order to make the model more general, it was decided to simulate only the sample with a 100% print density. As was the case in the experiment, the temperature is measured on the samples’ front surface while the rear surface is heated.

**Figure 5:**
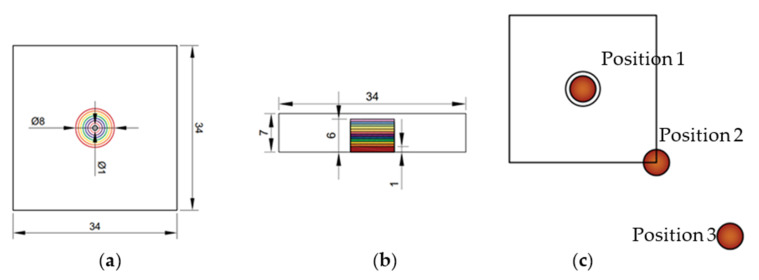
The parameterization of the numerical model. (**a**) Chosen defects diameters, (**b**) chosen defects’ depths, and (**c**) positions of the center of the heating source.

Our primary objective was to develop an effective, simplified model that does not completely replicate the whole original sample, but rather a subset of it. The utilization of numerical data obtained in this manner for network training enables the examination of samples with a variety of geometries and nearly any pattern of defects. Thus, the numerical model comprises a single defect located in the center of a small region. The model was suitably parameterized in order to collect data correlated with defects of various diameters and depths, as well as those positioned at varied distances from the heat source. The diameter of the defect changes between 1 mm and 8 mm (19 steps), the depth varies between 1 mm and 6 mm (15 steps), and the heat source is fixed in three positions: centered on the defect, on the corner of the modeled area plates, and beyond the plate at a distance of half its diagonal. We obtained a total of 855 distinct models. Figure 5 illustrates the parametrization procedure.

Calculations were performed using the Heat Transfer with Surface to Surface Radiation module, which uses the radiosity method to represent radiation on diffuse surfaces. In this case, we suppose that the only source of heat in the system is radiation [43,44]. In general, the following partial differential equation is used to solve the time−dependent heat transfer in solids problem:

(1) $$\rho C_{p}\frac{\partial T}{\partial t}+\nabla \left(q+q_{r}\right)=Q$$

where $\rho_{d}$ denotes the diffuse reflectivity, *G* is the irradiation, ϵ is the surface emissivity, and $e_{b}\left(T\right)$ is the power radiated across all wavelengths, according to the Stefan–Boltzmann law. The most critical aspect here is the heat flux introduced at the heated surface via radiation ($q_{r}$). The following formula is dependent on two significant variables: total irradiation and radiosity. The term “irradiation” refers to the entire incoming radiative flux induced by external energy sources, whereas “radiosity” refers to the sum of diffusively reflected and emitted radiation:

(2) $$J=\rho_{d}G+\epsilon e_{b}\left(T\right)$$

where $\rho_{d}$ denotes the diffuse reflectivity, *G* is the irradiation, ϵ is the surface emissivity, and $e_{b}\left(T\right)$ is the power radiated across all wavelengths, according to the Stefan–Boltzmann law. Radiative heat flow can thus be defined as the difference between incident and emitted radiation (radiosity and specular reflected radiation):

(3) $$q_{r}=G-\left(J+\rho_{s}G\right),$$

where $\rho_{s}$ is the specular reflectivity. Assuming the material behaves as an ideal grey body (as most opaque substances do), we finally obtain the correct formula for the radiative boundary condition by utilizing the radiative inward heat flux, ready to be included in Formula (1):

(4) $$q_{r}=\epsilon \left(G-e_{b}\left(T\right)\right)$$

Notably, the halogen lamp housing (see Figure 4), which is modeled as a conical, hollow object, is here regarded as a diffuse mirror, which is defined as a surface with zero emissivity. Diffuse mirror surfaces are frequently used as approximations for a well−insulated surface on one side, and for which convection effects on the opposite (radiating) side can be neglected. It is similar to a mirror in that it absorbs all incident radiation and then reradiates it in all directions. On a diffuse mirror boundary, the radiative heat flux is equal to zero.

### 3.2. Optimization Approach for Material Parameters Estimation

A critical component of this work is the correspondence between the numerical and experimental results. Naturally, as intended, the model used to train the network differs significantly from the real laboratory setup. Nonetheless, our objective is to obtain a strong correlation between the experiment and the model, as well as the maximum possible consistency of the results within the defects. Due to the fact that the numerical model employed a sample with a print density of 100%, the optimization procedure took into consideration only the experimental results obtained for sample S1 (100% print). The actual physical parameters of the sample may change slightly from the parameters reported in the literature for the PET−G material. Additionally, the modeled heat source is much different from the actual lamp. As a result, the heating power, which was adjusted to 1000 W during the experiment, should be treated as an unknown variable in the model. As we have observed, little variations in the aforementioned parameters result in significant variances in the time–temperature characteristics acquired for the numerical results. As a result, we propose employing the optimization method to accurately predict the model’s selected physical parameters [45,46,47].

Optimization was carried out in a hybrid Matlab/Comsol system. In Matlab, the optimization toolbox was employed. The optimization approach utilized a well−known pattern search (PS), which is a subgroup of direct search methods. Direct search is an optimization technique that does not require knowledge of the objective function’s gradient. The mentioned algorithms examine a group of points around the current position, looking for one where the objective function value is lower than the current point.

**Figure 6:**
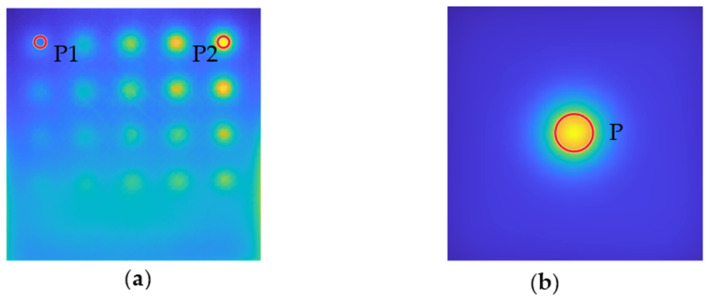
Localization of the areas taken under account in the optimization process. (**a**) Areas P1 and P2 localized on the chosen experimentally obtained thermogram, (**b**) area P localized on numerical thermogram.

In this study, the optimization parameters, normalized to [0,1], are: source power *P* (in the range 900–1500 (W)), specific heat capacity *Cp* (in the range 900–1500 (J/(kg⋅K))), density ρ (in the range 900–1500 (kg/m^3^)), and thermal conductivity *k* (from 0.1 to 0.5 (W/(m⋅K))). It should be highlighted that because the model shows a single defect at a time rather than a pattern of defects (like the actual sample does), the primary issue that has been neglected here is the effect of other defects on the background temperature distribution. We will demonstrate that, despite this significant simplification, our approach allows for effective network training on the numerical model. However, it is evident that we will not attain complete consistency between the numerical model’s background and the real sample’s background. As a result, this task is omitted in this optimization, and we instead focus on adjusting the time–temperature characteristics of the flaws. Figure 6 shows several points of interest. Region P1 contains a defect that is 1.4 mm deep and 7 mm in diameter, and area P2 contains a defect that is 4.2 mm deep and 7 mm in diameter. A comparable area was chosen based on numerical data (see Figure 6b), denoted by P. The minimized objective function calculates the sum of mean square errors (MSE) between the averaged time–temperature characteristics from selected areas of the experimental and numerical results:

(5) $$\;\mathrm{Objective}\;=\sum_{t=1}^{360}{\left(D_{P1\exp}\left(t\right)-D_{P1num}\left(t\right)\right)}^{2}+\sum_{t=1}^{360}{\left(D_{P2\exp}\left(t\right)-D_{P2num}\left(t\right)\right)}^{2},$$

where *t* denotes time, and *D_P*exp*_*(*t*)*_*,*_ D_Pnum_*(*t*)** are averaged time–temperature characteristics from chosen regions for experimental and numerical data, respectively.

**Figure 7:**
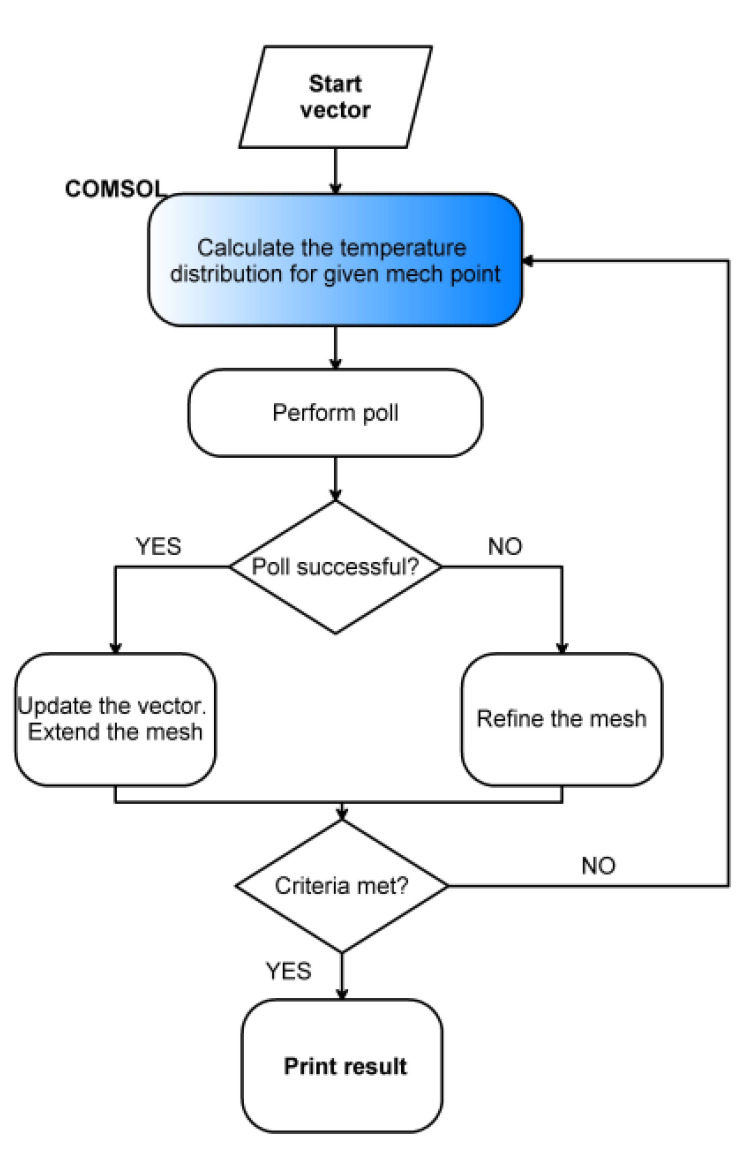
The chart flow of the optimization algorithm.

The starting point was chosen arbitrarily. At each optimization iteration, Comsol was used to calculate the number of models for the defect with a set depth and diameter while varying the optimization parameters mentioned previously. Each generated model was used to extract data from the P area. After averaging the data, they were compared to the relevant experimental data using the aforementioned objective function. Figure 7 depicts the algorithm diagram.

Table 1 compares the parameters specified in the literature for PETG material to those determined during the optimization method. As can be seen, the most significant change concerns the source’s power, which, as previously said, should be treated as an unknown.

**Figure 8:**
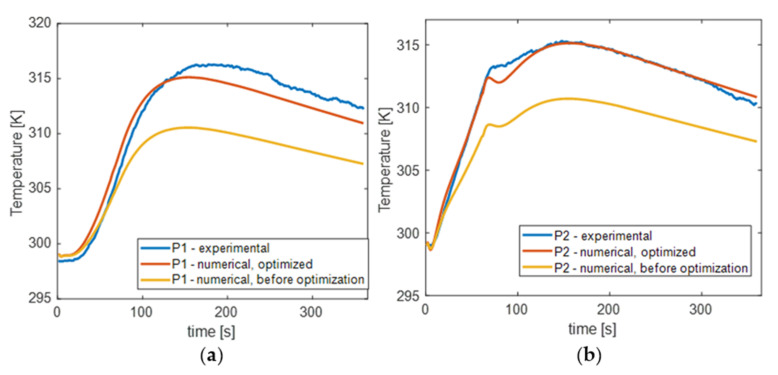
Averaged time–temperature characteristics from regions P1 and P2 compared with numerical data. (**a**) Comparison of the experimental (blue line) characteristic for the region P1 and numerical result: yellow line—before optimization, red line—after. (**b**) Analogical comparison for region P2.

To illustrate the optimization findings, the temperature–time characteristics of the numerical and experimental results were compared. The results from the P1, P2, and P regions were averaged for this purpose. Figure 8 depicts the outcome. As can be observed, the shape of the numerically produced curves is well−correlated with the experimental results, even prior to optimization, demonstrating the model’s sound physical assumptions. On the other hand, an improved quantitative agreement was also obtained following optimization. The largest difference between the numerical and experimental results for the P1 area was 1.86 K, which is slightly more than 10% of the reference value, whereas the maximum difference for the P2 area was 1.34 K, which is about 8% of the reference value.

### 3.3. The Comparison between Experimental and Numerical Results

**Figure 9:**
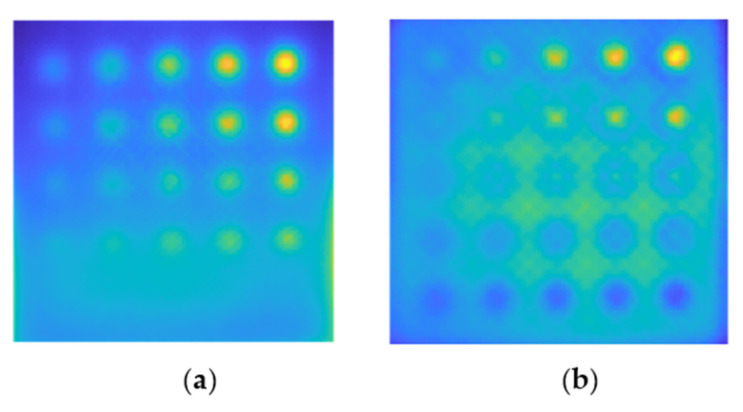
Exemplary experimental results for sample S1 (**a**) and S2 (**b**). The thermograms are obtained after the heating process—60 s of the observation.

Figure 9 illustrates the results of an inspection of real samples using the active thermography method. The thermograms presented are unprocessed and depict the temperature distribution on the surface of the tested samples after 60 s, i.e., after the heating process is complete. Figure 9a depicts a thermogram for the S1 plate (100% infill), and Figure 9b presents a thermogram for the S2 sample (with 30% infill). As can be seen, these results are rather dissimilar. Whereas defects are evident as hot spots in sample S1, smaller defects (starting with those with a diameter of 4.2 mm) are apparent as cooler spots in sample S2. It relates to the printing process itself−for items with a lower print density: internal structural features (such as holes) are produced with the support in the form of a layer with a 100% print density. These supports are readily evident in the cross−section shown in Figure 2. Due to the fact that these reinforced areas of the printout provide a stronger barrier to heat than their surroundings with only 30% infill, the thermal signatures of the flaws are visible as cooler areas.

**Figure 10:**
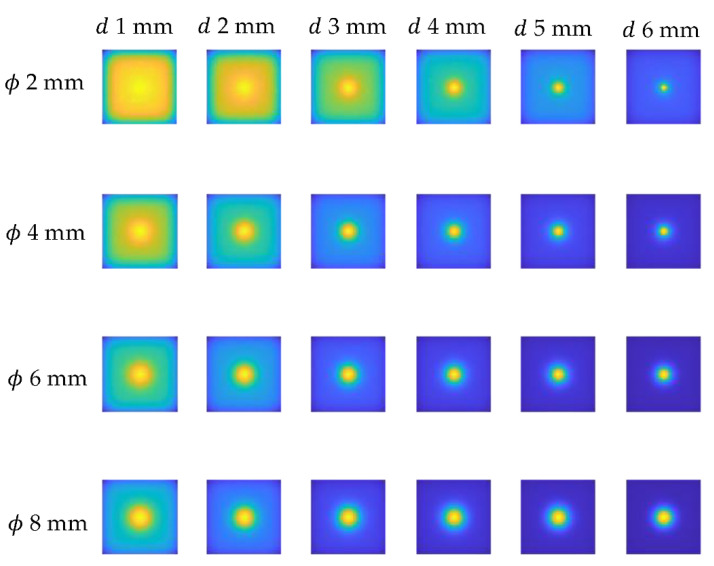
Exemplary numerical results for chosen defect diameter (ϕ from 2 mm up to 8 mm) and depths (*d* from 1 mm up to 6 mm).

As previously stated, optimization was performed on a sample with a 100% infill, implying that the numerical model’s results should be comparable to those obtained experimentally for this sample. Figure 10 illustrates the results for a variety of defects’ diameters and depths. As can be noticed, the flaw is visible in all the images as a warmer area. The qualitative difference between the experimental and numerical results is the distribution of the background temperature—it is notably more homogenous in the numerical data. As previously stated, this is a presumed effect due to the fact that the influence of other defects on the background temperature distribution has been neglected. The objective was to attain the highest degree of agreement feasible between numerical and experimental results within the defects, and hence within the indicated P1 and P2 areas.

## 4. Database Preparation

Creating a suitable database for neural network training is one of the most significant and fundamental tasks associated with the challenges of training neural networks. In this article, the database will be constructed solely from numerical data, and the effectiveness of the trained network will be evaluated using both numerical and experimental data, the latter of which will not be fed into the network training. As was indicated in the preceding chapter, a good correlation between numerical data and experimental data is required for the trained network to be effective in its application. This is a particularly difficult challenge in this study because we are working with a simplified model that only loosely corresponds to the real sample. It is also critical to process and select the data with care and precision. The method of data processing by eliminating the trend and normalizing will be provided in this section, as well as the process of data selection for the NN training.

### 4.1. Trend Removal Algorithm

As it is shown in the raw thermograms, presented in preceding sections, the localization of some defects may be hampered by a low signal−to−noise ratio. To address this issue, the trend elimination process should be used. Numerous image and data processing approaches can be used to eliminate the trend related to the heating characteristics of the source [48,49,50]. As previously stated, the assessed sample is a good thermal insulator; as a result, the indication of material inhomogeneity is fairly poor due to the low process dynamics. Additionally, a small defect size (ϕ = 1.4 mm), inhomogeneous sample thickness, defect localization at the edge, and lack of uniform heating contributed to the difficulty of detecting defects. Our earlier research [41] established that the algorithm based on curve fitting and Padé approximation is successful in this situation. This approach is based on the fact that the logarithmic polynomial of the given order is a good approximation of the curve for a one−dimensional time–temperature characteristic:

(6) $$\mathrm{Ln}(T\left(t\right))=\overset{N}{\underset{n=1}{\sum }}a_{n}\ln{\left(t\right)}^{n}$$

where *T(t)* denotes the temperature’s time evolution, *n* denotes the polynomial’s order, and *a* denotes the polynomial’s coefficients. The *N* value was empirically modified to match the data. The technique we propose replaces logarithmic functions with their Padé approximations. In particular, three Padé approximants of the following form were used to substitute the third−order logarithmic polynomial:

(7) $$\begin{array}{c} \ln(t)\approx \frac{2x-2}{x+1}\\ \ln{\left(t\right)}^{2}\approx \frac{{\left(x-1\right)}^{2}}{x}\\ \ln{\left(t\right)}^{3}\approx \frac{2{\left(x-1\right)}^{3}}{3x-1} \end{array}$$

**Figure 11:**
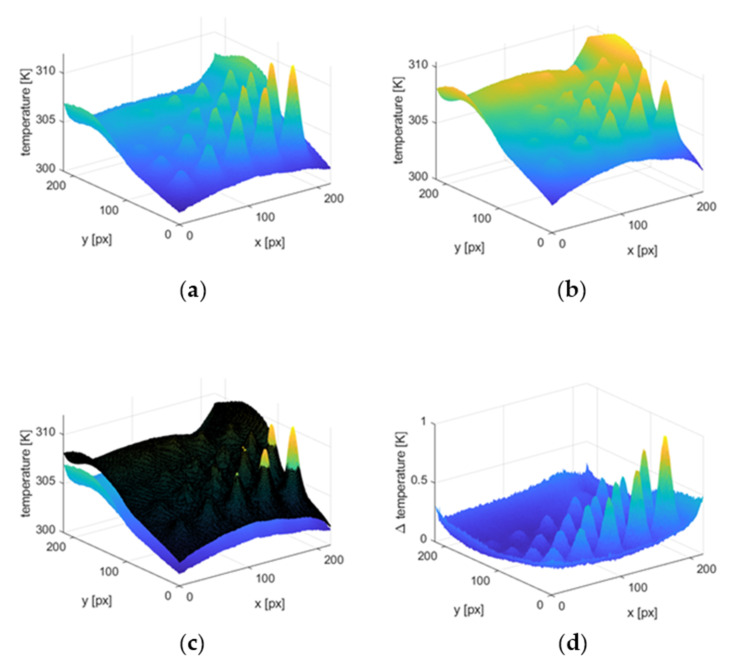
Procedure of the trend removal. (**a**) The original thermogram, (**b**) approximated thermogram, (**c**) overlay of the original image and its approximation, (**d**) the result of the subtraction of the approximation from the original.

According to our findings, using such an approach assures that the experimental results are more closely aligned with the approximation. The procedure of proposed trend removal technique is presented in Figure 11. In Figure 11a, the original thermogram is plotted, whereas in Figure 11b, its approximation is shown. The comparison between obtained results is shown as an overlay of the original thermogram with the approximation (Figure 11c). Subtracting the derived estimated characteristics from the initial ones is the last phase of the described technique. As a result, the trend is reduced, and the signal−to−noise ratio increases, revealing the thermal signatures of the defects, which is visible in Figure 11d.

**Figure 12:**
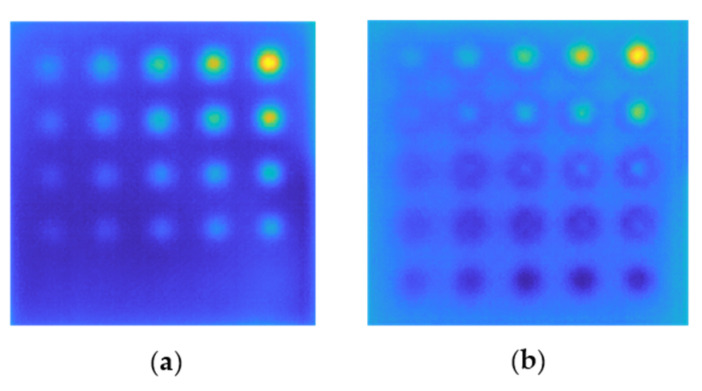
The result of the trend removal shown for the sample S1 (**a**) and S2 (**b**).

The results of trend removal for a sample with 100% infill (S1) and a sample with 30% infill (S2) are shown in Figure 12. As can be seen in the images, we were able to increase the visibility of defects in both samples. Only the smallest flaws with a diameter of 1.4 mm are invisible in sample S1, but all defects are evident in sample S2. Moreover, a further improvement has been made to the contrast between defective sections and the surrounding background for both samples.

**Figure 13:**
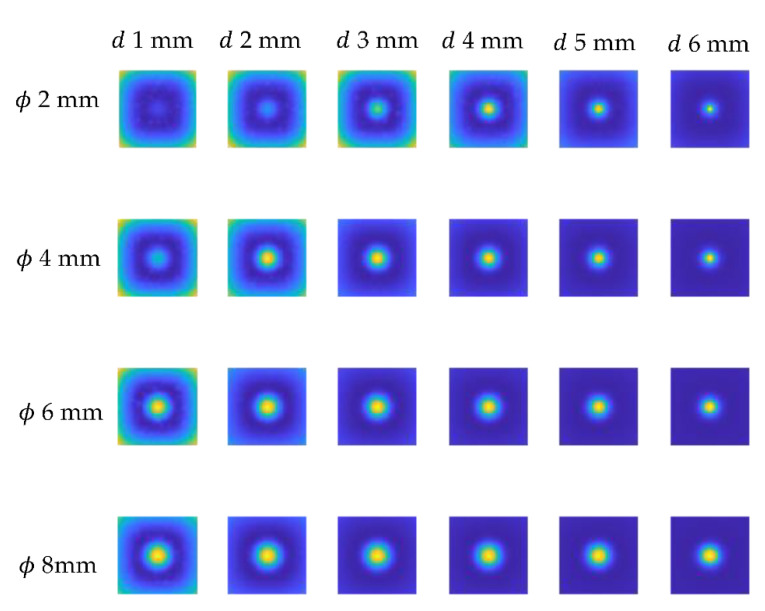
Exemplary results of the trend removal procedure executed on the numerical data shown for chosen defect diameter (ϕ from 2 mm up to 8 mm) and depths *(d* from 1 mm up to 6 mm).

In the numerical data, the algorithm enhances the visibility of minor flaws while eliminating background, which is shown in Figure 13, presenting exemplary results for the chosen defects’ depths and diameters. It should be noted, however, that the background leveling effect is visible in the immediate area of the defect, whereas there is visible heterogeneity in the image’s boundaries. This information must be considered while selecting the time–temperature characteristics for the network training base.

The final stage in preparing the results for the training database was to normalize the time–temperature characteristics extracted from each pixel of all the obtained numerical and experimental images. In this case, a z−score normalization was used, defined as follows:

(8) $$z\left(t\right)=\frac{x\left(t\right)-\mu }{\sigma }$$

where *x(t)* is characteristic value in time *t*, μ is the is the mean of the values of all the points in the characteristic, and σ is their standard deviation.

### 4.2. Selection of Characteristics for Training Procedure and the Final Structure of the Database

As previously stated, the primary purpose of this work was to construct a simplified and generalized numerical model, the outputs of which could be used to train neural networks, which would subsequently be able to evaluate experimental data. Because of this, the final database must include both numerical and experimental data, with the numerical data (divided into subsets training, validation, and testing, in the proportions 0.7:0.15:0.15) being used to train the network, while the experimental data will be used in their entirety during the network testing process after training.

It was assumed that the network’s purpose is to detect defects and so to classify the image into two categories: 0—without defects, and 1—with defects. As a result, two sets of training characteristics were collected: those related to the defect and those matching the image’s background. Overall, 300 characteristics from the defect location, and 10% of its surroundings were extracted from each image to create a database of defect−related features. The 10% increase in the defect area was intended to account for the defect periphery, which retains the defect area’s nature. For minor flaws with fewer than 300 pixels inside, Gaussian white noise with an SNR of 5 to 40 dB was added to the characteristics collected directly from the defect, and the resulting noisy characteristics were added to the set of defect−related features.

**Figure 14:**
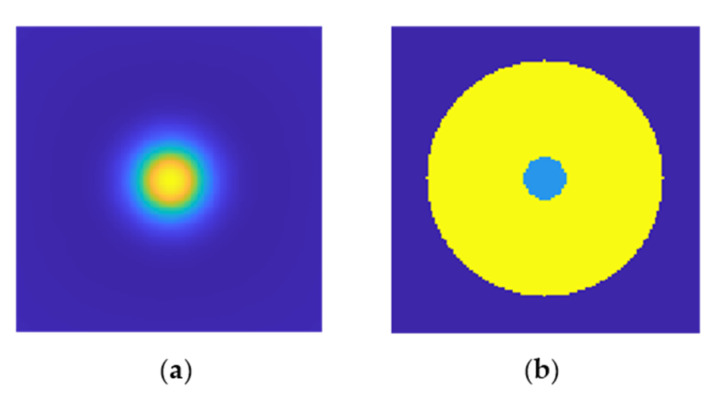
Critical areas of the image taken into account in the neural network database preparation. (**a**) The original image with clearly visible defect localization, (**b**) two critical areas: light blue—the defect area, and yellow—non−defect area.

In the case of the background characteristics, as previously stated, removing the trend from numerically obtained images improves the visibility of defects and the uniformity of the background around the defect, whereas the artifact in the form of an uneven temperature distribution on the image’s edges may be a source of disturbance, resulting in negative effects during network training. As a result, 300 random characteristics were added to the database of background characteristics for each image from the area surrounding the defect but excluding the image’s edges. Figure 14 denotes the areas from which both sets were extracted.

In general, as described in Section 3.1, 855 models were created as a result of parameterization (for different diameters and depths of defects and the position of the source). For each model, the sequences containing 360 images of the temperature distribution were collected. The database was populated using the time–temperature characteristics of 300 random pixels within the defect and 300 random pixels outside the defect from each obtained image sequence; thus, finally, the database, including 256,500 defect−related characteristics and 256,500 background−related characteristics, was created. As it was mentioned earlier, these data were randomly divided into training, validation, and test sets in a ratio of 0.7: 0.15: 0.15. The final training set includes 359,100 characteristics, 179,550 each for defects and the region without defects.

## 5. Defect Detection and Evaluation Procedure Based on Deep Learning

In contrast to more conventional feedforward neural networks, LSTMs feature feedback connections. As a result of this property, LSTMs may analyze whole data sequences (for example, time series) without having to consider each point individually, but rather by retaining crucial knowledge about past data in the sequence that can be used to aid in the processing of incoming data points. A consequence of this is that LSTMs are particularly adept at processing data sequences such as text, audio, and time series in general. In our case, it is certain that the presence of a defect has a significant impact on the complete set of time–temperature characteristics that have been investigated, both in the short− and long−term aspects. As a result, the employment of LSTM networks is completely warranted in this context.

### 5.1. LSTM Network Structure

**Figure 15:**
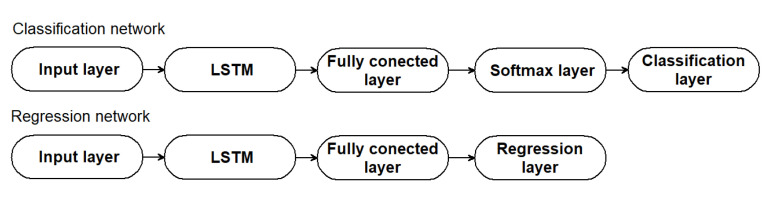
General architecture of LSTM neural networks.

In this study, we relied on the conventional MATLAB implementation of the LSTM network to complete our task successfully. These nets can be utilized in a sequence−to−label classification task as well as a sequence−to−sequence regression task, depending on the problem being performed. In all cases, the input and LSTM layers constitute the network’s core. A sequence input layer is responsible for bringing data from a sequence or time series into the network. An LSTM layer in a process of learning, establishes long−term dependencies between sequence data time steps. The classification network terminates with a fully connected layer, a softmax layer, and a classification output layer, whereas the regression network ends with a regression layer in place of the softmax and classification layers. Figure 15 presents the general structure of the LSTM networks.

The LSTM layer is where the learning occurs. LSTMs are distinguished from other forms of RNNs by their neuron structure, which is based on the gate mechanism. Three types of gates are recognized: forget, input, and output. The forget gate is used to erase unnecessary data. It accepts two inputs: new data and the output of the preceding cell. It filters these inputs using a sigmoid function and then multiplies them with the cell state. The input gate is in charge of updating the cell state. It operates similarly to the forget gate, in that it determines how much information should be retained using a sigmoid function. It uses the hyperbolic tangent function to produce a vector containing the required information. It then adds the beneficial information to the current cell state by combining the outputs of the sigmoid and tanh functions. The final output gate extracts valuable information based on the current state of the cell, the previous cell’s output, and any additional data received during the computation. This is accomplished by sending the cell state generated by the input and forget gates through a tanh function to generate a vector of values. After comparing the new data to the previous cell output, a sigmoid function is utilized to determine which values should be output. The output of the cell is created by combining the results of these two actions. The classification network is then terminated with a sequence of three classifying layers: fully connected, softmax, and classification output, all of which are used to forecast class labels. The fully connected layer is a one−dimensional flattened representation of the preceding layer, in which each neuron is connected to every neuron in the preceding layer, with each connection having its own weight. The softmax layer utilizes the softmax function to determine the probability that the input vector belongs to a particular class. In the regression network, the last two layers are replaced by a regression layer, computing the half−mean−squared−error loss for regression tasks.

**Figure 16:**
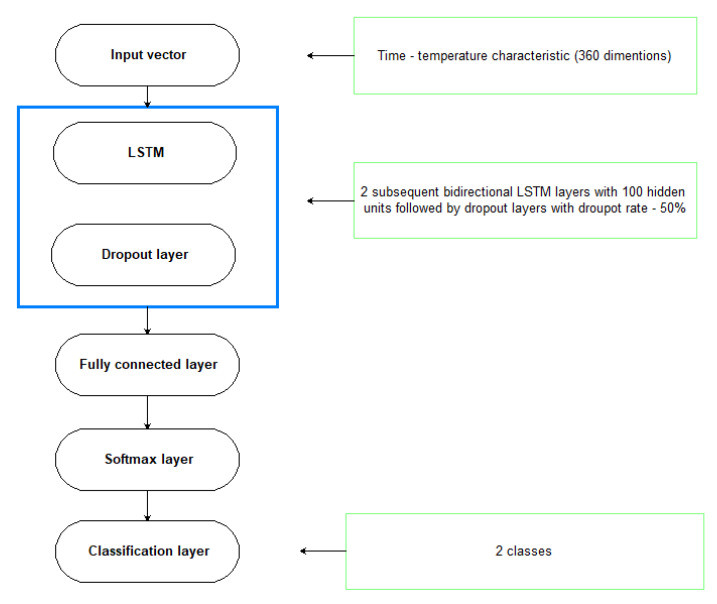
The architecture of the proposed neural network.

Prior to selecting the final LSTM network topology, preliminary experiments were conducted using LSTM layers, ranging in size from 30 to 500. Additionally, calculations were performed for various network depths utilizing one to five subsequent LSTM layers. Additionally, a dropout layer was incorporated into the network structure to mitigate the potential of an overfitting problem. The likelihood of deleting a given neuron from this layer in the subsequent round of the learning process was increased from 10% to 90%. Calculations were repeated ten times for each configuration, and the solution with the highest network accuracy value for the test set was chosen. The mini−batch size was also changed from 32 to 100, and finally, the value 80 was chosen. The maximum number of epochs was set at 100. Figure 16 depicts the final structure and configuration of the LSTM network obtained in a consequence of the preliminary trials.

### 5.2. Validation of the Network Performance

The generated neural network, trained on numerical data, was tested in two stages: in the first stage, the received network was fed with a test set randomized from the numerical data (15% of the data not used to the network training). This enabled the extraction of information on the confusion matrix and the coefficients associated with the accuracy, precision, and recall of neural networks. Additionally, all numerical data were fed to the network to demonstrate its effectiveness. The second stage involved loading the test data for both S1 (100% infill) and S2 (30% infill) to the network in order to determine its suitability for evaluating real, experimental data.

The following factors were used for evaluating the performance of the proposed network:

o True Positive (TP): expresses all the positive indications that were correctly classified as a positive (defect) class.
o False Positive (FP): expresses all the non−positive cases that were incorrectly classified as a positive class.
o True Negative (TN): expresses all the negative cases that were correctly classified as a negative (non−defect) class.
o False Negative (FN): expresses all the non−negative cases that were incorrectly classified as a negative class.

The four above−mentioned quantities can be given either in absolute numbers (in our case, these will be the numbers of the classified characteristics) or as a percentage. When combined, they generate a confusion matrix. Table 2 depicts the matrix of confusion for the numerical test set. The number of characteristics associated with the defect was equal to the number of characteristics associated with the background in this set (therefore, the test set had the same character as the set training the network). As can be seen, the proportion of false indications (false positives—FP and false negatives—FN) was less than 1%.

Furthermore, to evaluate the performance and robustness of the classification network, the following parameters were considered:

o Accuracy, defined as:
  $$\mathrm{Accuracy}=\frac{TP+TN}{TP+TN+FP+FN}$$
  This parameter represents the percentage of correctly classified indications.
o Recall, defined as:
  $$\mathrm{Recall}=\frac{TP}{TP+FN}$$
  This parameter refers to the proportion of correctly classified defects to all positive cases.
o Selectivity, defined as:
  $$\mathrm{Selectivity}=\frac{TN}{TN+FP}$$
  This parameter refers to the proportion of correctly classified non−defects to all negative cases.
o Precision, defined as:
  $$\mathrm{Precision}=\frac{TP}{TP+FP}$$

This parameter represents the proportion of correctly classified defects to all cases that the network classified as the defect class.

The mentioned parameters were calculated for the network tested on numerical data, and the results are gathered in Table 3.

**Figure 17:**
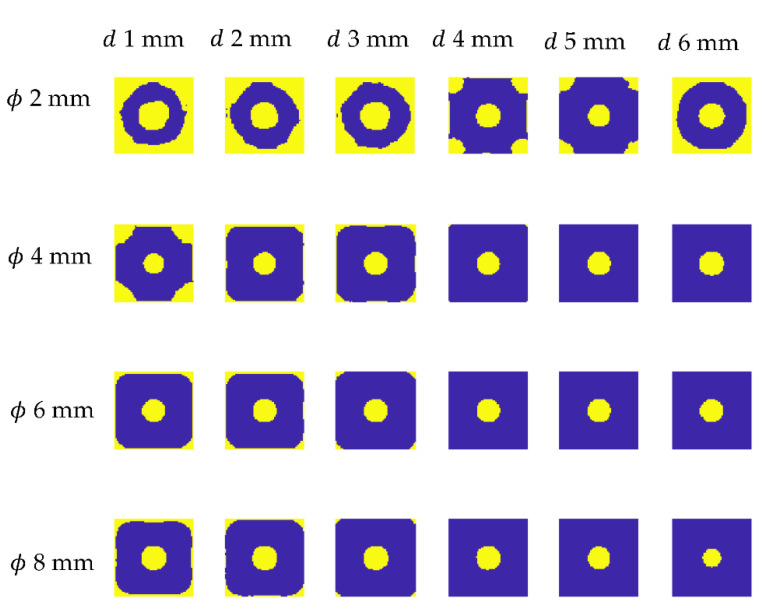
Exemplary results of the neural network classification executed on the numerical data shown for chosen defect diameter (ϕ from 2 mm up to 8 mm) and depths (*d* from 1 mm up to 6 mm).

To demonstrate the network’s operation on numerical data, the complete database was put into the network, which then classified all accessible time−frequency characteristics. Figure 17 illustrates the results for defects of various depths and diameters. Because the flaw is always centrally situated, it can be observed that the defect is localized in each of the cases illustrated. For smaller flaws, there is an issue with false positives at the image edges; this is clearly related to the thermogram characteristics (see Figure 13), where the signal−to−noise ratio was lowest for smaller defects and artifacts developed at the image edges. The second critical issue is defect oversizing: the network always delivers defects (i.e., pixels categorized as the defect class) with a diameter greater than the actual diameter. This is because the heat spread around the fault is relatively broad, rendering the area immediately surrounding the flaw indistinguishable from the actual defect for the network.

According to the obtained results, which include selected parameters characterizing the effectiveness of the network at a level close to 99% and actual indications of defects in the images obtained numerically, it is possible to conclude that the network was successfully trained for numerical data. We aimed to employ a trained, generic network for a specific purpose, such as detecting defects on real experimental data acquired from two separate samples. This was our primary goal in developing the network. The purpose of completing this work is to demonstrate that it is possible to use the generalized numerical model in a practical environment. To determine how the network handles experimental data similarly to how it handles numerical data, both databases (from two samples) were fed into the network input. To estimate the confusion matrix and selected network efficiency parameters, the pixels from experimental images were manually labeled and assigned to the classifications “defect”—1 and “not defect”—0. This result was attainable because all sample dimensions and locations in space during the experiment were precisely known. Dimensions have been translated from mm to px to ensure that all flaws are appropriately marked. As a result, a reference image was created against which the network output results were compared. It should be emphasized that, in contrast to the numerical test set, where exactly half of the data were characteristics related to defects and the other half were characteristics related to the background, there is no such relationship in the experimental data for obvious reasons—the total number of obtained characteristics related to defects was equal to 2316, while the total number of obtained characteristics related to the background was 50,687. As a result, very substantial percentage disproportions should be expected in the confusion matrices themselves.

Table 4 contains the confusion matrix for sample S1 (100% infill). As previously stated, significant percentage differences in the values of TN and TP are observed. To have a clearer view of the situation, absolute figures should be used here. For sample S1, the network successfully categorized 44,782 of the 50,687 (88.35%) characteristics associated with the background (non−defect class), and 1506 of the 2316 characteristics associated with defect areas (defect class) (which is 65.03%). Similarly to the numerical data, the network was precisely characterized using the four previously indicated parameters (Table 5). For the S1 sample, accuracy and selectivity coefficients values are above 90%; however, precision fell below 30%. The latter is inversely proportional to the quantity of false positives, which are many in comparison to true positives. This parameter is also substantially correlated with the low overall number of defect−related characteristics with respect to all available data.

A similar study was conducted on the S2 sample (30% print density), and the results obtained are comparable to those for the S1 sample. Table 6 contains the confusion matrix. Additionally, it is worth noting that the network categorized 44,485 background characteristics correctly (87.76%) and 1680 defect characteristics correctly (72.54%). All four characteristic parameters were recalculated, yielding an accuracy and selectivity of less than 90% and, once again, the lowest precision value of 21.88% (Table 7).

**Figure 18:**
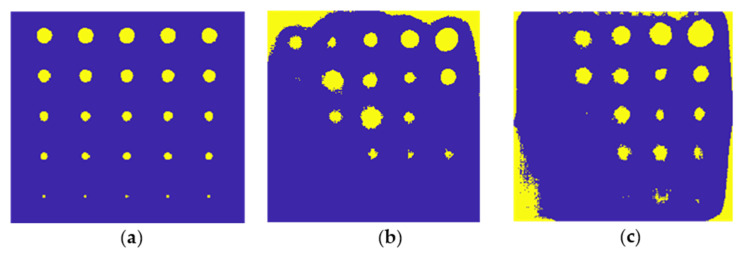
The results of the neural network classification executed on the experimental data. (**a**) The reference image with the defect and non−defect pixels labeled manually, (**b**) the result of classification for sample S1 and (**c**) S2.

The final stage of the results presentation is to depict the network’s classification of the experimental data obtained from both samples (S1 and S2) in the form of an image in order to demonstrate the network’s effectiveness in detecting defects. Figure 18 illustrates this visualization. In Figure 18a, an image with defects that have been manually marked is presented; thus, it serves as a reference image. Figure 18b illustrates the outcome for the S1 plate (100% infill). As can be seen, the majority of defects were correctly located, although the smallest defects (ϕ equal to 1.4 mm) were not indicated at all by the network. False positives occur near the image’s boundaries, which is attributable to the heterogeneity of the background, which persists even after the trend is removed. False positives have also been linked to an oversizing of some defects. Figure 18c illustrates the network classification result for sample S2 (30% infill). Once again, the majority of defects were correctly located; in this case, even some of the smallest defects were correctly detected, though their location is not as precise as in the case of major defects. Again, false positives emerge around the image’s periphery and around the faults due to an oversizing effect.

## 6. Conclusions

Employing a generic numerical model as a basis, the presented study gives a thorough examination of the feasibility of using active infrared thermography in conjunction with signal analysis assisted by deep neural networks to detect defects in objects created using 3D printing technology. Most importantly, we sought to demonstrate that a very general model can be used to train the LSTM network and, as a result of this, gain a tool that can be used to assess the experimental findings in a real setting. As a result of the model’s generalization, it is possible to classify a wide range of experimental data, as it was demonstrated with two samples for verification: one with 100% and another with 30% infill. Despite the fact that the experimental results for the two samples were qualitatively different, the network trained on the generalized model produced good and comparable classification results in both cases, detecting most of the present defects.

Additionally, this study demonstrated how critical it is to properly prepare the model and then the data for the network training foundation in this sort of research. The numerical model was constructed as a parameterized collection of models, with the material parameters of the simulated sample being optimized in the first step using the experimental data. This strategy resulted in a stronger correlation between the numerical and experimental results; however, it should be noted that this adjustment was conducted only on a few randomly chosen areas of the sample with 100% infill (S1). In the second stage, all data—numerical and experimental—were submitted to a trend removal and normalization process. The presented trend−removal approach, which is based on the Padé approximation, performs well in the presence of minor flaws in materials with poor thermal conductivity. The final stage of preparing the training base was to select time–temperature characteristics; for this purpose, critical areas—the area of the defect and the area surrounding the defect—were determined, excluding areas near the images’ edges, which were particularly susceptible to artifacts caused by non−uniform heating.

For the classification challenge, a sort of regression network called an LSTM network was utilized. This approach was necessitated given the character of the training data. The time–temperature characteristics provide information about the heating and cooling processes that occurred at a particular point in the sample. These functions will typically be distinct for points in the defect region and points in the background region. The existence of a defect alters the shape of the entire characteristic, and analyzing it both globally and locally (short−term changes between successive points of the characteristic) offers additional information. The LSTM network permits the investigation of signal temporal sequences while accounting for both long− and short−term effects, resulting in improved feature extraction.

As a consequence of the study reported here, a network, trained solely on the numerical data generated from the generalized model was created. It was shown that the resulting network performs well when classifying experimental data. The resultant network was evaluated on numerical data in the first step, yielding extremely high values for the network’s standard metrics—accuracy, sensitivity, recall, and precision (all above 98 percent). Although the findings were significantly poorer for the experimental data, it should be recalled that the data were distorted by noise, and the results are not entirely compatible with the generalized numerical model. Nonetheless, good findings were achieved for both samples; the majority of faults were confined, and the false positives that reduced the precision parameter’s value were found on the image’s perimeter and around the defects.

The research provided here focused on a single form of defect—holes—and simplified the geometry of the object. We intend to expand our study in the future to include additional sorts of structural defects, such as internal defects. Additionally, a regression work will be added to the classification task in order to continually detect the geometrical dimensions of the flaws and obtain a quantitative evaluation.

## Figures and Tables

**Table 1:** Comparison between the important parameters before and after the optimization process.

	Power [W]	Heat Capacity $C_{p}\;[J/\left(kq\cdot K\right)]$	Density $\rho \;\;[kg/m^{3}]$	Thermal Conductivity $k\;\;[W/\left(m\cdot K\right)]$
Literature data ^1^	1000	1242	1200	0.225
Optimized values	1363	1110	1339	0.222

^1^ From PET−G Technical Data Sheet—Fiberlogy.

**Table 2:** The confusion matrix for numerical data.

	Predicted Class		
		Non−defect	Defect
**Actual class**	Non−defect	49.37%	0.63%
	Defect	0.08%	49.92%

**Table 3:** The network parameters computed for the numerical data.

Accuracy	Recall	Selectivity	Precision
99.30%	99.84%	98.75%	98.76%

**Table 4:** Confusion matrix for the experimental data (sample S1).

	Predicted Class		
		Non−defect	Defect
**Actual class**	Non−defect	88.50%	7.31%
	Defect	1.21%	2.98%

**Table 5:** Network parameters computed for the experimental data (sample S1).

Accuracy	Recall	Selectivity	Precision
91.48%	71.07%	92.37%	28.93%

**Table 6:** Confusion matrix for the experimental data (sample S2).

	Predicted Class		
		Non−defect	Defect
**Actual class**	Non−defect	84.25%	11.36%
	Defect	1.20%	3.18%

**Table 7:** Network parameters computed for the experimental data (sample S2).

Accuracy	Recall	Selectivity	Precision
87.43%	72.57%	88.12%	21.88%
